# Scale, Skill‐Mix, and Access Implications of the Production of Appointments by Primary Care Practices in England

**DOI:** 10.1002/hec.70064

**Published:** 2025-11-20

**Authors:** Tianchang Zhao, Rachel Meacock, Matt Sutton

**Affiliations:** ^1^ Health Organisation Policy & Economics School of Health Sciences. University of Manchester Manchester UK; ^2^ Centre for Health Economics Monash University Monash Australia; ^3^ Melbourne Institute: Applied Economic & Social Research The University of Melbourne Melbourne Australia

**Keywords:** GLM, GP appointments, primary care skill‐mix, production function, scale

## Abstract

Primary medical care has traditionally been provided by small organisations. Recent policy developments in many countries have encouraged larger practices in the hope of benefiting from increasing returns to scale, but there is little research evidence to support this. Using monthly data from 6149 primary care practices in England between August 2022 and July 2024, we applied a Generalized Linear Model with a logarithmic link and Poisson distribution to examine the relationship between staffing levels and appointment volumes. At the median level of administrative staffing, the estimated marginal productivity of doctors and other clinical staff on total appointments are 223 and 152 per month, respectively. The marginal effects of all types of staff on appointment volumes increase with staffing levels. We plot the isoquant and isocost curves at the median level of production and examine the implications of our findings for skill‐mix and patient access. The current ratios of doctors to other clinical staff and nurses to Direct Patient Care (DPC) staff are lower than cost optimal, though this is less of an issue for larger practices who benefit more from DPC roles. Additional clinical staff improve patient access more when employed in larger practices.

## 1 Introduction

The relationship between productivity and the scale of healthcare providers has long been a topic of research. A large body of literature regarding optimal hospital size, often measured by bed capacity, suggests that larger hospitals are generally more efficient as they benefit from lower average costs and improved specialization, though excessively large hospitals might suffer from diseconomies of scale due to increased management complexity and lower quality of care (Carr and Feldstein 1967; Dranove 1998; Giancotti et al. 2017; McCallion et al. 2000). In contrast, the understanding of scale in non‐hospital services, particularly primary care practices, remains less developed.

Family doctor partnerships, unlike hospitals, operate on a much smaller scale, face low fixed costs, involve less advanced technology, and provide more homogeneous services (Bodenheimer and Pham 2010; Shi 2012; Starfield et al. 2005). These structural and operational differences are significant when considering returns to scale. Hospitals, with their high fixed costs and reliance on specialized technology, are more naturally positioned to benefit from economies of scale as they spread these costs over a larger patient base. Other unique challenges in primary care practices may also limit their ability to benefit from scale. Many family doctors are business owners and issues such as moral hazard in larger teams and financial incentives may create conflicts between profit maximization and patient care priorities (Gaynor and Gertler 1995; Hausman and Le Grand 1999). These tensions may undermine productivity gains that could otherwise arise from scaling up. Additionally, while the relatively homogeneous nature of primary care services might make them more amenable to standardization and efficiency improvements, the expansion of partnerships often introduces managerial complexities. Larger practices require more coordination and governance, and involve more complex resource allocation, which may offset potential gains from scaling. These factors create ambiguity in the relationship between practice size and productivity, distinguishing primary care practices from hospitals in their potential ability to benefit from scale.

In recent years, the NHS in England has undertaken a strategic push to promote larger‐scale provision in primary care to enhance care quality, accessibility, and efficiency (Forbes et al. 2020; NHS and England 2016). Since 2014, NHS guidance has encouraged practices to operate at scale through various collaborative models, including networks, federations, super‐partnerships, multisite organizations, and mergers, seeking to achieve “better outcomes”, “better partnerships”, “better value”, and to be “better for the workforce” (NHS England 2014). By early 2018, 55% of practices in England served populations of over 30,000 people through such arrangements (Forbes et al. 2019). These reforms aim to address key challenges facing primary care, such as workforce shortages, growing patient demand, and disparities in access and care quality. Expanded scale allows for the workforce to be used more flexibly, with roles and responsibilities distributed across a broader team to meet patient needs effectively. Larger‐scale partnerships are also expected to achieve cost savings through economies of scale in administrative and business functions, and the ability to invest in information and analysis infrastructure that smaller practices may find unaffordable (NHS England 2014).

Despite the nationwide drive towards larger family doctor partnerships and collaborative organisational models, the empirical evidence on the relationship between sizes of primary care organizations and performance remains inconclusive (Damiani et al. 2021). Increases in list size have been associated with mixed changes in clinical outcomes such as management of long‐term health conditions, hospital admissions, emergency department attendances, and prescribing behaviour (Kovacevic et al. 2023). Larger partnerships have also been linked to lower continuity of care and worse patient‐reported outcomes (Forbes et al. 2020; Gravelle et al. 2022). Conversely, primary care networks appear to perform better than individual practices on certain service delivery measures including vaccination uptake, care plan completion, referral behaviour, and screening uptake (Kovacevic et al. 2023). Large‐scale primary care organisations may also be more capable of implementing standardised safety and quality processes and adopting new technologies, both of which can lead to quality and efficiency improvements (Pettigrew et al. 2019). Whilst the evidence on many performance metrics is therefore mixed, the impact of practice size on a core primary care output, the production of clinical appointments, remains underexplored. To our knowledge, only one study has analysed the effect of size on the production of consultations. This was undertaken in the context of Danish primary care and used the registered population rather than staff inputs as the measure of size (Olsen et al. 2013).

In addition to the number of staff, the mix of staff working in practices will also determine their productivity. New clinical professionals have been introduced into the primary care workforce in England to relieve workload pressures on physicians and nurses, while specific reasons to hire new roles depend on types of practitioners and practice managers (McDermott et al. 2022). Motivations include increasing appointment availability, freeing up GP time, and improving the alignment between patient needs and the care provided. Some practices are also using the new roles as a pragmatic short‐term solution to GP shortage. Recent studies have examined the relationship between skill‐mix changes and primary care outcomes, finding that the implementation of skill‐mix can be challenging due to the inherent complexity of primary care caseloads. The introduction of new roles was associated with worse patient experience and higher overall expenditure (Francetic et al. 2022; McDermott et al. 2022). However, to our knowledge, only two attempts have been made to date to estimate the production of clinical appointments in the English context. Due to data availability, one of these studies (Williams et al. 2023) was undertaken for higher‐level groups of practices (Clinical Commissioning Groups) and therefore the effects of practice size could not be estimated. The other study examined the relationship between various factors and appointment volumes at practice level, but labour inputs were assumed to have linear effects and to be independent from each other (Zhao et al. 2023). While it is anticipated that larger family doctor partnerships may yield benefits through increasing returns to scale with enhanced resource allocation and streamlined operations, no empirical evidence has been generated on the production of the key primary care output, clinical appointments and its relationship to numbers of staff.

This paper aims to fill these gaps by estimating flexible relationships between different labour inputs and the production of primary care appointments at practice level. Following the recent publication of data on appointment volumes at practice level for all general practices in England, we estimate the production function for primary care appointments and assess the implications for practice scale and skill mix. Using practice workforce as the inputs, we quantify the direct contribution of each type of staff to the provision of appointments, conditional on the size of practice measured by staffing levels and controlling for a measure of population needs. In addition to total appointment volumes, we pay particular attention to the marginal effects in the production of *timely* appointments, defined as occurring within 2 days of booking. Timely access is a key determinant of patient satisfaction and may impact the whole healthcare ecosystem by preventing avoidable use of some hospital services (Parkinson et al. 2021; Rosano et al. 2013). Using isoquant and isocost curves we also identify the most cost‐effective skill‐mix combinations. Finally, unlike most previous studies, we consider the number of people registered with the practice as an output of production, reflecting the capitation payment system in England which pays practices for providing a year of care to each of their registered patients. By analysing the effects of labour inputs on numbers of registered patients, total appointments, and timely appointments, this study offers new insights into access to primary care and its scalability.

## 2 Methods

### 2.1 Variables

#### 2.1.1 Outputs

We examine seven different measures of primary care output, six of which capture appointment volumes of various types. We first analyse the total volume of clinical appointments, and then a subset of these which take place within 2 days of being booked (timely appointments). We then further categorize both of these into appointments with a physician and appointments with other practice staff.

We obtained data on practice‐level appointment volumes from August 2022 to July 2024 (the earliest month covered by the data series to the latest data published at the time of analyses) (NHS Digital 2024c). The data series records the number of booked and attended appointments each month, distinguishing between those delivered by a GP or by other clinical practice staff, and the time between booking and the appointment taking place. The data is collected directly from GP systems and the majority of practices use either the EMIS or the TPP GP system. There is no significant difference in the characteristics of practices using different GP systems (Kontopantelis et al., 2018). A small proportion of appointments recorded with unknown times between booking and occurrence are pro‐rated using the proportions that are known, following the Office for National Statistics (ONS) methodology for computing primary care output (ONS 2021).

We also consider the number of registered patients as our seventh measure of a practice's productivity, indicating the number of patients for which a practice is contracted to provide care. Because some types of patients are expected to generate more workload than others, we use the normalised need‐weighted number of patients, following the weighting used by NHS England for resource allocation (NHS Digital 2024b; NHS England 2023). We consider list size as an output rather than input for two reasons. First, patient list size is limited by practice productivity, not the other way round. Practices can stop accepting new registrations if they do not have extra capacity to look after more patients, and patients can switch to other practices if their current practice is not able to provide sufficient care. To measure how effective this mechanism is, depending on the availability of alternative providers, we include rurality and the Herfindahl‐Hirschman Index (HHI) of practice concentration in the analysis. Second, although utilization depends on the demand for services, it is reasonable to assume that most practices are currently operating at full capacity given the ongoing primary care crisis in the UK (Gerada 2021; Razai and Majeed 2022). On the aggregate level, the shortage of FTE qualified GPs was estimated at 4200 in 2022 and projected to rise to 6700 by 2024 (Shembavnekar et al. 2022). Since 2015, the number of FTE GPs has decreased by 1,115, while the average number of patients per GP has increased by 320 (BMA 2024c). Given this national workforce shortage, most practices are unlikely to be able to fully adjust their staffing to meet rising demand. This is evidenced by the sharp decline in patient satisfaction in recent years and by the public's view that improving access to GP appointments should be the NHS's top priority (Taylor et al. 2025). Any further increase in registered patients alone would not increase total appointments provided in this context of full capacity operation.

#### 2.1.2 Input Variables

We obtained data on the full‐time equivalent (FTE) numbers of qualified GPs, GP trainees (registered junior doctors in general practice speciality training), nurses, other direct patient care professionals (DPC), and administrative staff employed at each practice in each month from NHS Digital's General Practice Workforce data series (NHS Digital 2024a).

Administrative staff and two groups of clinical labour inputs are included in the production function of each of the outputs, corresponding to the two categories of appointments produced. Administrative staff, doctors (including both qualified and trainee doctors) and other clinical staff (including nurses and DPC professionals) are the inputs for total practice appointments, 2‐day appointments, and registered patients. Since GPs cannot produce “appointments with other practice staff” and vice versa, only qualified GPs and GP trainees are included separately as inputs for “appointments with a GP”; and only Nurses and DPCs are included separately as inputs for “appointments with other practice staff”. Administrative staff enter both functions as the third input, as they support the production of all appointment types. The outputs and the corresponding input variables are listed in Table 1. The boldfaced variables are considered the main clinical input in the production of the respective outputs.

**TABLE 1:** Outputs examined, corresponding input variables, and control variables.

Outputs	Inputs	Control variables
Total appointments Two‐day appointments Weighted patients	Administrative staff **GP (qualified and trainee)** Non‐GP staff (Nurse and DPC)	Need index^a^ Time fixed effects HHI^a^ Urban or rural location
GP appointments GP 2‐day appointments	Administrative staff **Qualified GP** GP trainee	
Other appointments Other 2‐day appointments	Administrative staff **Nurse** DPC	

^a^ HHI is only included in the model for weighted patients, while the need index is not included in the model for weighted patients, as the patient numbers are already weighted.

#### 2.1.3 Control Variables

The practice level needs index, calculated as the normalised need‐weighted population divided by the number of registered patients, is included in the six production functions for appointment volumes. As we want a measure of the average level of workload associated with the appointments provided at a practice, we do not adjust for the Market Forces Factor element of the resource allocation formula when weighting the population (NHS England 2021). Two additional factors, the HHI and practice rurality, are included in the model of weighted patients. The HHI measures the level of competition in the area (measured at Lower‐layer Super Output Area (LSOA)) in which the practice is located using the sum of squared market shares of each practice (Rhoades 1993). The HHI for area l is calculated as:

$${HHI}_{l}=100\times \sum_{i=1}^{n}{\left(\frac{\text{patients}\;\text{in}\;\text{the}\;\text{LSOA}\;\text{registered}\;\text{with}\;{\mathrm{practice}}_{i}}{\text{total}\;\text{patients}\;\text{in}\;\text{the}\;\text{LSOA}\;l}\right)}^{2}.$$

and each practice *i*’s HHI is assigned based on the LSOA in which their main surgery is located.

Lower HHI values indicate a less concentrated and hence more competitive market. The HHIs are computed using the numbers of registered patients as of July 2024, when the latest practice level LSOA distribution of registered patients was published (NHS Digital 2024b). We used share of registered patients, rather than share of services, to measure competition for two reasons. First, in England, practice income is primarily determined by list size, adjusted for patient age and gender, rather than appointment volume. Second, patients can only receive care from the practice with which they are registered. While practices can apply to temporarily close their lists if operating at capacity, they are otherwise required to accept all eligible patients within their catchment area without discrimination (BMA 2024a). Rurality is obtained from the 2023 NHS Payments to General Practice dataset (NHS Digital 2023a). In a supplementary analysis we also estimate the functions including additional practice characteristics: dispensing status, contract type, and the Care Quality Commission (CQC) rating, to account for potential variations in unobservable factors that potentially have an impact on productivity, such as managerial styles, financial incentives, and the quality of inputs. Practices with an overall performance rating (as in the March 2024 release of CQC data) of “requires improvement” or “inadequate” are classified as having a low CQC rating, and “outstanding” performance as high CQC rating (Care Quality Commission 2024). Although all three of these covariates are not technically constant, there was close to no within‐practice variation observed within the study period, and they are therefore considered time‐invariant in the model. We used the July 2024 release of the CQC data in the analysis, and the dispensing status and contract type are taken from the 2023 Payment to General Practice data (NHS Digital 2023a).

Our dataset covers all primary care practices in England, with the following exclusions: practices that were not included in all 24 releases of the Appointments in General Practice data series, that is, those opened or closed during the study period; inactive practices and those with an appointment rate of less than one appointment per registered patient per year (NHS Digital 2023b); practices with fewer than 1000 registered patients, as these may be in the process of opening or closing; and practices with missing characteristics or workforce data. Of the 6198 practices included in the July 2024 release of GP appointment data, 6149 were included in our analysis, covering 62.95 million registered patients or 99.3% of all registered patients in England as of July 2024 (NHS Digital 2024b).

### 2.2 Statistical Methods

#### 2.2.1 Model Specification and Estimation

The seven outcome variables we examine are technically count variables, therefore strictly positive and highly right skewed. To avoid negative predicted values and large sample‐to‐sample variation due to skewed error terms, we adopt a Generalized linear model (GLM) (Deb et al. 2017). The input variables are assumed to be exponentially linked to the outputs, that is, $E\left[Y_{it}|X_{it}\right]=\exp\left(X_{it}^{\prime }\beta \right)$, where $Y_{it}$ is the output produced by practice i in period t and $X_{it}$ a matrix of input variables. Equivalently, the equation can be rearranged into $\ln\;E\left[Y_{it}|X_{it}\right]=X_{it}^{\prime }\beta$, making the model log‐linear.

Following previous literature that has modelled outputs in health care using a production function approach, our model specification incorporates squared and cubic terms of the inputs and interactions between each pair of inputs, making the model highly flexible (Francetic et al. 2022; Preyra and Pink 2006). We chose a cubic functional form over the widely adopted transcendental logarithmic (translog) production function for two reasons. First, as we are interested in the marginal effects of the input variables on the number of appointments produced, using a translog model would require retransformation to the natural scale from the logarithmic scale, which is complex if the errors are heteroskedastic (Manning 1998). Second, a considerable proportion of practices, especially the smaller‐scale ones, do not employ any GP trainees and/or DPC staff, meaning that the values of these inputs are zero which cannot be log‐transformed.

We create 23 period dummy variables to account for seasonality and time trends. Our model of appointment volumes produced by practice i in period t is specified as follows:

$$\ln\;E\left[Y_{it}^{j}|X_{it}\right]=\sum_{m=1}^{3}\sum_{q=1}^{3}\gamma_{mq}x_{imt}^{q}+\sum_{\begin{array}{c} m_{1},m_{2}\in \left(1,2,3\right)\\ m_{1}\neq m_{2} \end{array}}\eta_{m_{1}m_{2}}x_{im_{1}t}x_{im_{2}t}+\delta Need_{i}+\sum_{t=2}^{24}\tau_{t}period_{t},$$

where j is the type of output and m the type of staff. The first term on the right‐hand side denotes the cubic polynomial of the three workforce inputs, $x_{m}^{q}$ and their coefficients $\gamma_{mq}$, where q is the exponent of the term. The second term captures interaction effects of numbers in each staff group. The outcome variables are assumed to be generated from a Poisson distribution, although this choice of distribution family in a GLM estimation has no impact on the consistency of the estimated parameters, only on the efficiency of estimation (Deb et al. 2017). The parameters are estimated using maximum likelihood optimization and standard errors are clustered at the practice level.

#### 2.2.2 Marginal Effects

We then compute the marginal effects, or marginal production, of each staff input variable on the respective output at the 25^th^, 50^th^, and 75^th^ percentile levels of two measures of practice size: administrative staff, and of the main clinical staff input variable (identified for each model in Table 1), that is,

$${\text{marginal}\;\text{effect}}_{im}=\frac{\partial E\left(Y_{i}|X_{i}\right)}{\partial x_{m}}{│}_{{\left(V_{Q_{i}}\right)}_{i=1}^{3}},$$

where $V_{Q_{i}}$ is the variable used as the measure of practice size evaluated at the three quantiles. Given the cubic form of the production function, the average marginal effect of an input is:

$$\frac{\partial E\left(Y_{i}|X_{i}\right)}{\partial x_{1}}=\left[\gamma_{11}+\gamma_{12}x_{1i}+3\gamma_{13}x_{1i}^{2}+\eta_{12}x_{2i}+\eta_{13}x_{3i}\right]\exp\left(X_{it}^{\prime }\beta \right),$$

where β is the concatenation of all coefficients, and the other variables are held at median levels to represent an average practice. We test the null hypothesis that the difference between marginal effects evaluated at 25^th^ and 75^th^ percentiles of practice size are significantly different from zero using a Wald test. The marginal effects are also evaluated at the 10^th^ to 90^th^ percentiles of the size variables and plotted. Increasing marginal effects indicates that an extra unit of staff input is estimated to contribute more to the output as the size of practices increases.

#### 2.2.3 Isoquant and Isocost

Using the estimated production function, we plot the isoquants for different levels of output, that is, sets of clinical staff combinations that are required to provide a certain number of appointments or to provide care for a certain number of patients. We then use the isoquant curves and the average unit costs of the two types of clinical staff inputs to obtain the cost‐minimizing ratios of staff (Gravelle and Rees 2004). Note that nurses and DPCs are considered as one main type of input in the total appointment model as their products are grouped together into one category of “appointments with other practice staff” by NHS Digital in the dataset. At any level of output, the minimum cost combination of inputs occurs when the slope of the isoquant – the marginal rate of technical substitution (MRTS) between a pair of clinical staff inputs – is equal to the ratio of the costs of inputs, that is,

$$MR{TS}_{1,2}^{j}=-\frac{\partial Y^{j}}{\partial x_{1}}/\frac{\partial Y^{j}}{\partial x_{2}}=-\frac{P_{x_{1}}}{P_{x_{2}}},$$

where $P_{x_{m}}$ is the unit cost of the input. Table 2 shows the unit costs used for each type of staff (K. C. Jones et al. 2024). We use unit costs of GPs which exclude travel or qualification costs, and the unit cost of Non‐GP staff is calculated as a weighted average of the unit costs of Nurses and DPC staff.

**TABLE 2:** Annual unit costs of each type of staff input.

GP^a^	Non‐GP^b^	Qualified GP	GP Trainee^a^	Nurse^c^	DPC^d^
£239,555	£97,473	£279,399	£105,471	£100,024	£95,022

^a^ Estimated cost of a GP, which includes trainee GPs. Calculated as the weighted average of qualified GP and GP trainee unit costs, where the unit cost of a GP trainee is the weighted average of trainees of different grades. (BMA 2024b).

^b^ Weighted average of the unit costs of nurse and DPC.

^c^ The unit costs of different pay bands weighted by the number of nurses in each band. (NHS Health Careers 2022).

^d^ Direct patient care staff are costed at the same level as a band 6 GP practice nurse (K. C. Jones et al. 2024).

### 2.3 Supplementary Analyses

To assess the robustness of our estimates, we conducted the following supplementary analyses.

#### 2.3.1 Omitted Variable Bias

The input variables may be correlated with unobserved practice characteristics, such as management efficiency, labour market factors, the level of altruism of the practice owners, and the roles and productivity of staff. All of these may have direct impacts on appointment volumes and bias the estimated coefficients. To confirm that our results are not strongly biased by such factors, we have re‐run our main model controlling for additional variables including the level of socioeconomic deprivation in the local area, the dispensing status of the practice, the contract type of the practice, and six quality ratings given by the Care Quality Commission (safety, effectiveness, caring, responsive, well‐led, and overall). We also controlled for region and sub‐region location to account for geographical differences in workforce availability, patient demand, and service delivery models.

#### 2.3.2 Simultaneity

Appointments are outputs of staff inputs and, by definition, cannot *cause* changes in staffing levels. However, a potential concern is that demand for appointments, though not directly measurable, may influence staffing decisions, introducing simultaneity bias. To address this, we have assumed that demand exceeds supply across all practices, such that practices are unable to fully adjust their workforce to meet patient demand.

To support this assumption, we examined responses to the General Practice Patient Survey from 2019 (the last pre‐pandemic wave) and 2023 (midpoint of our study period), focusing on the question: “Overall, how would you describe your experience of making an appointment?” A significant decline in satisfaction suggests that demand has outpaced supply over time. As a robustness check, we re‐estimated our main models excluding practices where patient satisfaction with making appointments did not decline, as these may be exceptions to the assumption.

Additionally, we reran the main analysis using lagged workforce inputs (specifically, staffing levels from the previous month [t‒1] and the previous quarter [t‒3]), which are less likely to be results of demand at time t.

#### 2.3.3 Staff Heterogeneity

We assessed the sensitivity of our findings to greater staff heterogeneity by modifying the specification of input variables in three ways. First, we re‐estimated the GP Appointments model by disaggregating the “qualified GP” category into “GP partners” and “salaried GPs,” while keeping GP trainees and administrative staff as separate input variables. Larger practices tend to employ more salaried GPs, who are younger and less experienced than GP partners, on average. Additionally, salaried GPs may be less motivated than partners, who have ownership stakes in the practice. These factors may influence GP productivity and thus appointment volumes.

Second, we have re‐run the Other Appointments model, splitting nurses into band 6 nurses and band 7 nurses. Band 6 nurses include practice nurses and dispenser nurses and the majority of general practice nurses are in band 6 (NHS 2024; NHS Digital 2024a). Band 7 nurses include Advanced Nurse Practitioners, Nurse Specialists, and Extended Role Practice Nurses. A small number of nurse partners and other advanced practice nurses are also included in this band. Although the nurse partners are in band 8, we assume their productivity in terms of appointment provision are the same as band 7 nurses. There are fewer than 200 band 5 trainee nurses in England, who are not included in this analysis as an input variable to keep the model parsimonious. DPCs were not further disaggregated due to the lack of a nationally standardised definition and the diversity of roles included in this category.

Third, we re‐estimated the main model using salary‐weighted workforce inputs to account for differences in average staff costs and, by proxy, experience and productivity levels. Non‐GP clinical staff were weighted relative to the salary of a band 6 nurse, and salaried GPs were weighted relative to GP partners based on their average salaries. Locum GPs were assigned the same weight as salaried GPs, as we assume the average productivities of the two types are comparable.

## 3 Results

### 3.1 Descriptive Statistics

Table 3 presents descriptive statistics summarising the mean and distribution of practice average appointment volumes, weighted patients, and five groups of practice staff inputs. An average practice in England provides 4709 appointments per month, just under half of which are delivered by GPs. Of all appointments produced, 51.22% took place on the same or next day following booking. This is much higher for GP appointments, of which 66.51% were same or next day appointments. There is more variability between practices in the provision of 2‐day appointments than in total appointments.

**TABLE 3:** Descriptive statistics for output and staffing input variables (24‐month practice average), *N* = 6194.

	Mean	SD	p10	p25	p50	p75	p90
Output variables							
Total appointments	4709	3321	1666	2582	3990	5890	8446
Total 2‐day	2412	1814	783	1235	1992	3041	4453
GP appointments	2180	1530	761	1175	1843	2775	3886
GP 2‐day	1450	1103	450	734	1185	1870	2704
Other appointments	2407	1963	705	1143	1914	3068	4548
Other 2‐day	963	984	194	357	682	1209	2002
Weighted patients	9376	6347	3630	5328	8050	11,521	16,091
Workforce FTE							
Administrative	11.887	9.559	4.133	6.134	9.609	14.559	21.121
Total GP	5.719	4.327	1.389	2.503	4.811	7.858	11.02
Qualified	4.341	3.131	1.308	2.165	3.698	5.68	8.068
Trainee	1.379	1.726	0.000	0. 000	0.889	2.249	3.529
Other practice staff	5.234	5.382	0.961	1.96	3.751	6.773	10.718
Nurse	2.627	2.643	0.480	0.978	1.922	3.452	5.387
DPC	2.607	3.223	0.117	0.729	1.65	3.364	5.921

*Note:* The mean number of GP appointments and appointments with other staff do not add up to the mean total appointments due to a small proportion of appointments recorded without information on the type of staff.

**FIGURE 1:**
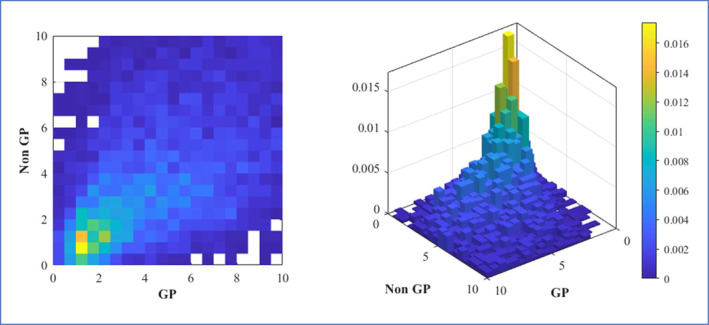
Joint distribution of practice level numbers of FTE GP and non‐GP staff. The colour scale represents the proportion of all practices having the particular combination of GP and non‐GP staff.

The distributions of workforce inputs exhibit considerable variation. Over 25% of practices did not employ any GP trainees in the 24‐month period, and 25% of practices employed fewer than one FTE nurse or DPC. All variables shown in Table 3 are strongly positively skewed. The joint distribution of the 24‐month average practice‐level numbers of GPs (including trainees) and other clinical staff is shown in Figure 1. A large proportion of practices employ one to three FTE GPs and a similar amount of other clinical staff, although the variation between practices is substantial, with some practices having ratios of staff groups over 5:1 either way.

#### 3.1.1 Production Function and Marginal Effects

The estimated production functions are reported in Supporting Information S1: Appendix 1 (Table A1). Since we used a logarithmic link function in the GLM, the conditional means of the outputs are in exponential form and the coefficients can be interpreted as the approximated percent changes of appointment volume corresponding to a one‐unit change in the respective input variables (Deb et al. 2017). The overall average effect of each input variable, including the quadratic terms and interactions, is calculated as

(1) $$\begin{array}{c} \%\Delta y=\left(\exp\left\{\sum_{q=1}^{3}\gamma_{1q}\left[{\left({\bar{x}}_{1}+1\right)}^{q}-{\bar{x}}_{1}^{q}\right]+\sum_{m=2,3}\eta_{1m}{\bar{x}}_{m}\right\}-1\right)\times 100, \end{array}$$

where $x_{1}$ is the variable of interest and $x_{2},x_{3}$ are the other two input variables. On average, a one FTE increase in GP inputs is associated with an approximately 5.83% increase in total appointment volumes and a one FTE increase in non‐GP staff is associated with a 3.66% increase. However, these effects on total appointments are smaller than those of an additional FTE qualified GP and GP trainee inputs on GP appointments (13.25% and 6.43% respectively), or those of an additional FTE nurse and DPC on appointments with other practice staff (11.76% and 4.90% respectively), as a result of substitution between the two main groups of staff in the production of total appointments.

Table 4 reports the marginal products of a unit increase in each of the three staff categories on the seven output variables for practices of different sizes, as measured by the staffing level of administrative staff or the main type of input for the respective output variable (GP, qualified GP, or Nurse as outlined in Table 1). The marginal effects are evaluated at the 25^th^, 50^th^, and 75^th^ percentile of staffing levels observed in our sample. For example, at a practice with the median level of administrative staff, employing an extra FTE GP is estimated to increase total monthly appointments by 223 and 2‐day appointments by 131. The marginal effects of GPs are higher than those of non‐GP staff for total appointments, 2‐day appointments, and weighted patient. The difference is particularly large for the marginal effects on weighted patients, with the marginal effect of a GP at least four times that of non‐GP staff. The standard error for each estimated marginal effect is reported in Supporting Information S1: Appendix 1 Table A2.

**TABLE 4:** Marginal effects of input variables on monthly appointment volume, evaluated for practices of different sizes measured by staffing levels of either administrative or the main type of staff input.

Practice size as measured by:	Administrative staff			GPs		
	25th	50th	75th	25th	50th	75th
Total appointments						
Admin	132.56	138.14	144.62	133.62	143.82	154.96
Non‐GP	138.99	152.37	171.13	118.99	138.19	164.19
GP	208.03	223.39	242.91	219.12	220.51	217.88
Two‐day appointments						
Admin	58.92	62.91	68.85	69.94	74.77	79.57
Non‐GP	75.94	78.29	81.42	48.49	57.66	70.60
GP	125.31	130.69	137.72	118.34	118.73	116.50
Weighted patient						
Admin	299.73	323.72	357.08	319.85	344.76	371.75
Non‐GP	100.52	105.65	110.58	50.40	70.26	101.16
GP	416.26	452.68	501.00	442.56	442.80	433.35
**Practice size as measured by:**	**Administrative staff**			**Qualified GP**		
Total appointments with GP						
Admin	47.50	47.07	45.82	40.56	46.73	54.47
GP trainee	88.51	97.74	111.02	98.24	110.10	123.01
Qualified GP	210.09	224.40	243.04	207.63	228.01	250.02
Two‐day appointments with GP						
Admin	33.23	33.16	32.61	29.54	33.48	38.22
GP trainee	70.46	76.72	85.38	71.74	81.07	92.00
Qualified GP	126.98	135.91	147.53	130.00	139.01	147.43
**Practice size as measured by:**	**Administrative staff**			**Nurse**		
Total appointments with other practice staff						
Admin	79.03	82.21	83.78	63.35	71.86	85.67
DPC	83.27	95.90	114.16	84.18	95.10	112.64
Nurse	195.34	226.28	271.64	254.26	261.32	263.03
Two‐day appointments with other practice staff						
Admin	28.12	29.23	30.16	22.81	25.87	30.94
DPC	39.89	44.02	49.64	36.07	40.82	48.66
Nurse	86.13	97.47	114.05	102.72	107.82	113.13

*Note:* The ordering of the sizes of marginal effects are not the same with the two measures of practice size because the practices differ in the ratio of GPs to administrative staff which they employ. This means that practices at the 25^th^, 50^th^ and 75^th^ percentile of administrative staff are not in general the same as those at the 25^th^, 50^th^ and 75^th^ percentiles of GPs.

**FIGURE 2:**
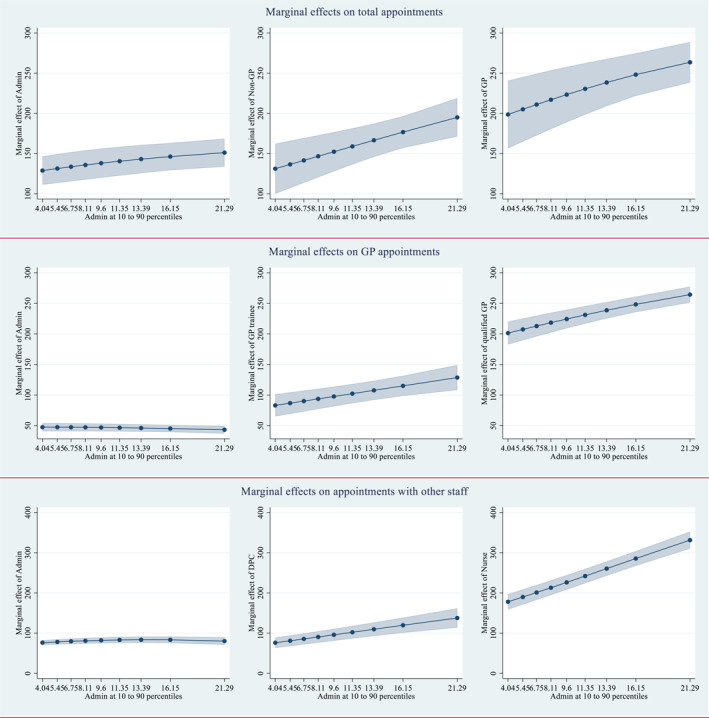
Marginal effects on total appointments evaluated at the 10^th^ to 90^th^ percentile of Admin staffing level.

The differences between marginal effects evaluated at 25^th^ and 75^th^ percentiles are tested against zero and the results are shown in Supporting Information S1: Appendix 1 Table A3. The marginal effects estimated at the 10^th^ to 90^th^ percentile of Admin staffing level are plotted in Figure 2. In general, the marginal product of an input increases consistently with practice size measured by staffing level, with the exception of administrative staff inputs, whose effects on appointment volumes do not vary significantly with the administrative staffing level. The other exception is the marginal product of GPs (including trainees) evaluated at different levels of GP staffing. This likely occurs because practices with higher numbers of total GPs have much higher proportions of trainees, the productivity of whom is lower than for qualified GPs and who require supervision from qualified GPs. For example, for practices in the lowest 25^th^ percentile of total number of GPs, 2.85% of those are trainees, whereas for those in the highest 25^th^ percentile the proportion is 29.27%.

Estimating the combined effect of both qualified GPs and trainees, as in the first models (outlined in row one to three in Table 1), overlooks the substitution between the two groups whose marginal products are very different. When evaluated separately in the model of GP (and 2‐day GP) appointments, the marginal products of both trainee and qualified GPs on GP appointments increase consistently with practice size (Table 4, Total appointments with GP). The substitution problem does not occur in the estimation of appointments with other staff which combines Nurses and DPC staff, whose marginal productivities are also very different, because the ratio between Nurse and DPC staff employed is not strongly correlated with either of our measures of practice size.

Alternatively, to examine the relationship between scale and the productivity of GPs which have a particularly large effect on the number of registered patients, we also calculated the marginal appointment rates, defined as GP's marginal production of appointments divided by the marginal effect on weighted patient numbers. For practices at the 25^th^, 50^th^, and 75^th^ percentile of FTE GPs, the marginal appointment rates are 0.495, 0.498 and 0.503 per weighted patient per month. They are 0.267, 0.268 and 0.269 per weighted patient for 2‐day appointments. The finding that these numbers are increasing with practice size indicates that an additional GP employed at a larger practice provides more appointments compared to an additional GP employed by a small to medium sized practice. The additional capacity could lead to better access for all existing registered patients at the larger practice.

However, it is worth noting that the ratio of the marginal effects of an extra GP on timely appointments to that on total appointments is decreasing with practice size, as shown in Table 5 (Admin 25th: 0.602, 50^th^: 0.585, 75^th^: 0.567; GP 25^th^: 0.540, 50^th^: 0.538, 75^th^: 0.535). The lower proportion of timely appointments indicates that while the larger practices benefit from scale in terms of total appointments produced, patients have to wait longer on average when the scale of the practice increases.

**TABLE 5:** Marginal appointment rates and the ratio of marginal effect on 2‐day versus. total appointments.

	Administrative staff			GPs		
Practice size as measured by:	25th	50th	75th	25th	50th	75th
Marginal appointment rate^a^						
GP	0.500	0.493	0.485	0.495	0.498	0.503
GP 2‐day	0.301	0.289	0.275	0.267	0.268	0.269
Two‐day/total ratio^b^						
GP	0.602	0.585	0.567	0.540	0.538	0.535

^a^ Marginal appointment rate is calculated as marginal effects on appointments/marginal effects on weighted patients.

^b^ Two‐day/total ratio is calculated as marginal effects on 2‐day appointments/marginal effects on total appointments.

The marginal effects of an additional FTE GP on the number of GP appointments are higher when evaluated at higher staffing levels. An additional qualified GP employed by a practice with a relatively high level of administrative staff (75^th^ percentile of all practices) is estimated to lead to 243 more appointments, compared to 210 if employed by a practice with a relatively low level of administrative staff (25^th^ percentile of all practices). The marginal effects of DPC and Nurses on appointments with other practice staff also increase with practice size. The stepwise difference between the estimated marginal productions can be as high as approximately 20%, (e.g., the marginal effects of non‐GP on total appointments at 75^th^ is 22% higher than at the 50^th^ percentile of GP). Using the effects estimated at the median staffing level (50^th^ percentile) as baseline, those evaluated at the 75^th^ percentile can be over 30% higher than that at 25^th^ percentile.

Plots of the marginal effects of each input evaluated at the 10^th^ to 90^th^ percentile of staffing levels are included in Supporting Information S1: Appendix 1 (Figure A1, Figure A2, Figure A3). Most plots show clear positive slopes, indicating larger practices benefit more from additional clinical staff. There are a few exceptions with large confidence intervals, making the change in marginal effects as practice size increases less clear the increasing proportion of GP trainees. Administrative staff demonstrate relatively constant marginal effects on appointment volumes but increasing effects on registered patients.

#### 3.1.2 Isoquant and Isocost Curves

**FIGURE 3:**
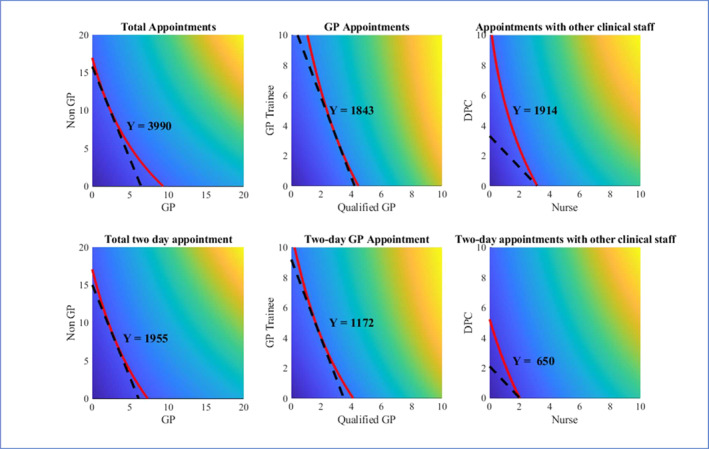
Estimated appointment volumes evaluated at the median level of administrative staff. *X* and *Y* axes indicate the FTE numbers of the respective type of clinical staff. The colouring scale in each plot represents relative values within the plot and is not standardised across all six plots, i.e., dark blue in the bottom left corners represents low numbers of appointments and bright orange on top right corners represents high values, but the ranges of estimated appointments differ on the six plots. The isoquant curves for median levels of appointments in each category are shown in red and the dashed lines are the corresponding tangent isocost curves given the unit costs of the two respective types of clinical staff.

**FIGURE 4:**
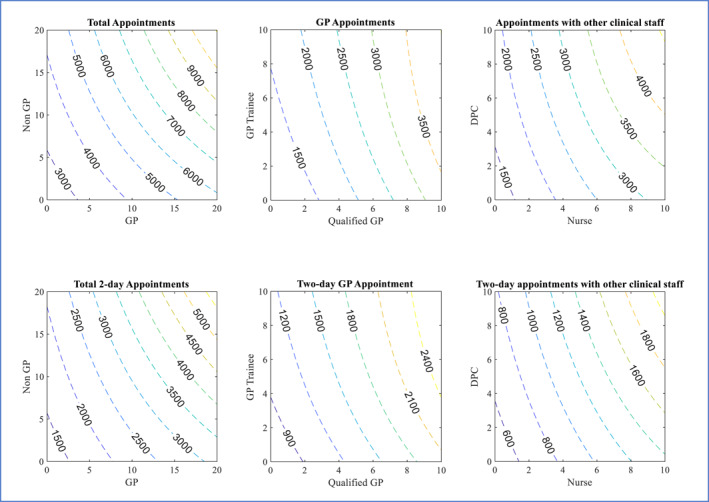
Estimated isoquant curves at different levels of monthly production corresponding to values of input variables. Numbers on the isoquant curves are the monthly levels of respective output variables.

Figure 3 shows the predicted productions of the six categories of appointment types at different levels of staff inputs, holding all other variables at their median values. An isoquant curve is plotted at the median level for each output variable. The lowest possible isocost curve that is tangent to the isoquant curve is also plotted according to the ratio of unit costs for the two inputs. The tangent point indicates the combination of staff inputs that can produce the median level of appointments with the lowest total cost, assuming that the services produced by both types of inputs are identical. Figure 4 shows contour plots of the six production functions. The isoquant curves in the contour plots are more densely located towards the top right corner of the plots where input levels are the highest, meaning fewer extra staff are required to produce more appointments as the overall staffing level increases.

Given the unit costs of GP and non‐GP staff inputs, the slope of the isocost line in the total appointments plot is −2.46 and it is tangent to the isoquant curve at values of two FTE GP and approximately 11 FTE non‐GPs (Figure 3). The cost of inputs on this isocost line is £1.55million per annum, representing the theoretically lowest possible cost required to produce the median level of appointments. The tangent point suggests a very low “optimal” GP to non‐GP staff ratio for a typical practice to produce a median level of appointments under the assumption that appointments provided by GPs are identical to those provided by a non‐GP staff. This assumption is, however, unlikely to hold in reality as estimates suggest that at least 30% of the tasks performed by GPs require the expertise of a fully trained GP and therefore cannot be delegated to other practice staff (Carlisle 2015; Richardson and Maynard 1995). The median GP to non‐GP staff ratio observed in our sample is 1.22, implying a MRTS of approximately −1.3, and requiring approximately 5.5 GPs and 4.5 non‐GPs to produce the median level of appointments at a cost of £1.75million. Assuming the average production per FTE clinical staff at a practice is constant regardless of clinical skill‐mix, we have the following equations:

$$\frac{2\cdot n_{\mathrm{GP}}\cdot v_{\mathrm{GP}}+11\cdot n_{\mathrm{non}-\mathrm{GP}}\cdot v_{\mathrm{non}-\mathrm{GP}}}{5.5\cdot n_{\mathrm{GP}}\cdot v_{\mathrm{GP}}+4.5\cdot n_{\mathrm{non}-\mathrm{GP}}\cdot v_{\mathrm{non}-\mathrm{GP}}}=\frac{£1,551,313\;}{£1,753,785},\;\;$$

$$2\cdot n_{\mathrm{GP}}+11\cdot n_{\mathrm{non}-\mathrm{GP}}=5.5\cdot n_{\mathrm{GP}}+4.5\cdot n_{\mathrm{non}-\mathrm{GP}}=3990,$$

where $n_{m}$ and $v_{m}$ represent the average production of one FTE staff of type m and the implied “value” or “importance” assigned by the practice to an appointment delivered by a staff of type m, respectively. The equations imply that the ratio between the “value” of a GP appointment to non‐GP appointments to a practice is approximately 1.32, that is the practices' willingness to pay for an “appointment with a GP” is approximately 1.32 times that for an “appointment with other practice staff”.

Similarly, the unit costs of Nurses and DPCs suggest that at the median level of output observed in our sample, it is most cost‐efficient to employ only nurses and almost no DPC staff. This is because Nurses demonstrate much higher productivity in terms of the number of appointments produced but only cost 5.3% more than DPC staff. However, as shown in Figure 4, as we move towards the top right corner of the graph, where the size of workforce and appointment volume are both higher, the isoquant curves become more convex. As a result, the isoquant and isocost curves at higher levels are tangential at points closer to the diagonal, indicating a lower “optimal” Nurse to DPC ratio. These results suggest that larger practices can benefit from a more balanced skill‐mix of nurse and DPC staff, while small to medium practices may not have the scale required for DPC staff to be most productive, as many of the DPC roles tend to be more specialised. We do not analyse substitution between qualified GPs and GP trainees as the latter is a temporary phase and there is a strong lagged correlation between the two inputs.

**FIGURE 5:**
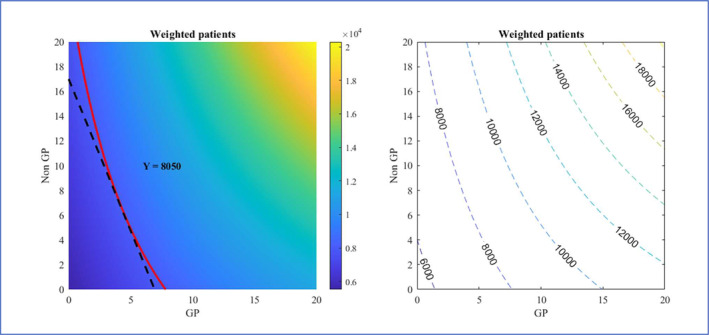
Estimated productions, isoquant curves and isocost curve at the median level of production, examining weighted patients as the output.

Figure 5 plots the estimated relationship between GP and non‐GP staff inputs and the weighted number of registered patients. While the isoquants appear to be approximately symmetric with respect to the diagonal in the total appointment contour plots, indicating GP and non‐GP staff are almost perfect substitutes in the production of appointments, the number of patients for whom a practice can provide care depends largely on the number of GPs, especially for smaller practices. The lowest isocost curve tangent to the median production isoquant curve intersects with the vertical axis at 17 and has a slope of −2.45, that is, non‐GP=17.2−2.45GP and the tangent point is GP=4.3,non‐GP=6.4. The isocost line suggests that a GP to non‐GP ratio of approximately 0.67 would be the most cost‐efficient to provide care for the median level of registered patients.

As the inputs increase, two observations can be made from the contour plot in Figure 5. First, the isoquants become less negatively sloped and more symmetric with respect to the diagonal at higher levels of the two input values, meaning the MRTS is getting closer to one on the diagonal and the two inputs are becoming perfect substitutes as practice size increases. The tangent point with the isocost curve moves further to the top left from the diagonal and the optimal GP to non‐GP ratio decreases. Second, the isoquants are getting closer to each other, meaning that the increase in clinical staff required to provide care to another 2000 patients is lower than that required by the previous 2000 patients. This effect would be much stronger if the number of administrative staff also increases, which would likely be the case in reality, instead of being held constant at the median level as we have done in producing the plots.

### 3.2 Supplementary Analyses

#### 3.2.1 Omitted Variable Bias

Inclusion of additional covariates (area deprivation, dispensing status, contract type, CQC ratings, and regional controls) did not materially change the estimated coefficients or marginal effects of the input variables. This suggests that the original model is unlikely to suffer from substantial omitted variable bias. Full regression results and marginal effect estimates are presented in Appendix 2 (Table A4, Figure A4).

#### 3.2.2 Simultaneity

Between 2019 and 2023, average patient satisfaction with overall experience and with appointment booking declined significantly, from 0.851 to 0.740 and from 0.704 to 0.573, respectively (Table A5). In our sample of 6149 practices, 5281 (86%) reported lower overall satisfaction and 5056 (82%) reported lower satisfaction with making an appointment. These patterns suggest that most practices are struggling to meet increasing demand, supporting our assumption that supply of appointments remains below demand. When we restricted the analysis to practices with declining satisfaction in appointment access, the estimated marginal effects were slightly higher, while the positive relationship between marginal effects and practice size persisted (Figure A5). Models using lagged input variables (t−1 and t−3) yielded results highly consistent with the main analysis. Regression results (Table A6) and marginal effect plots (Figure A6) are provided in Appendix 3.

#### 3.2.3 Staff Heterogeneity

Disaggregating “qualified GP” into “GP partners” and “salaried GPs” produced results comparable to the main specification. Marginal effects of GP trainees remained nearly identical, while the original estimate for “qualified GPs” fell between those of salaried GPs and GP partners, with the former showing slightly higher effects (Figure A7, Figure A8). In both models, the marginal effects of all GP inputs increased with practice size.

In the Other Appointments model, distinguishing between band 6 and band 7 nurses did not materially alter the results (Figure A9, Figure A10). Band 7 nurses were estimated to have higher marginal effects than band 6 nurses, and the increase in marginal effects with practice size was steeper for band 7 nurses. DPC staff estimates remained largely unchanged. In all cases, marginal effects continued to rise with practice size.

Finally, re‐estimating the model with salary‐weighted workforce inputs led to somewhat higher marginal effects for GPs and slightly lower effects for non‐GP staff. Excluding GP trainees or weighting them at half the salary of GP partners increased the estimated marginal effects for GPs but did not alter the overall trend. The marginal effects for the other two staff categories remained stable. Marginal effect plots from all three sets of analyses are provided in Appendix 4 (Figure A11, Figure A12, Figure A13).

## 4 Discussion

### 4.1 Summary

This paper contributes to the literature on primary care productivity, efficiency, and patient access, by modelling the production function of primary care practices in England for the first time using newly available data on practice‐level appointment volumes. For an average practice, an additional FTE GP is estimated to increase appointment volumes by 5.83%, and the marginal effect of an additional FTE GP is 223 appointments per month for an average practice with the median level of administrative staff. We found strong positive correlations between the marginal product of appointments associated with each type of staff and practice size measured by either level of administrative staff or the main type of clinical staff, suggesting that additional staff members increase appointment volumes by more when added to larger practices than smaller practices. Sufficient administrative support is critical to unlock the productivity potential of clinical staff and to improve patient access.

### 4.2 Comparison With Existing Literature

Earlier literature establishing that economies of scale exist in the administration of primary care have mostly focused on the impact of scale on costs instead of production capacity, due to the lack of available data capturing a consistent and comparable measure of primary care output (Giuffrida et al. 2000; Neri et al. 2022). As expected, without including the patient list size as an input variable, which is highly correlated with the workforce variables included in this study, the estimated coefficients of staff inputs on appointment volume are overall higher than shown in a previous study (Zhao et al. 2023). Additionally, the degree that practices benefit from scaling up is likely to be higher following the introduction of health information technologies and electronic clinical decision support systems, whose adoption and effective implementation are strongly correlated with larger practice size (De Rosis and Seghieri 2015; Ford et al. 2021; Police et al. 2010). While evidence from secondary care settings suggests that there is an optimal hospital size and that some existing hospitals fall outside of this range, we did not find evidence of decreasing marginal productivity of primary care services over the current range of practice size, suggesting that practices could benefit from increasing in size beyond that currently observed in England (Giancotti et al. 2017). Our results therefore provide support for the NHS strategy of increasing the scale of operation in primary care (Forbes et al. 2020; NHS England 2016).

Using the production function and the unit cost of each type of workforce input, we identified the cost optimal staffing ratios required to produce the median level of appointments, finding that the current average non‐GP to GP ratios (0.91) and nurse to DPC ratios (approximately 1) are much lower than ideal. This indicates that for an average practice to increase the number of appointments provided to patients, it would be more cost‐efficient to focus on the recruitment of nurses instead of GPs or DPC roles. As a large literature has shown that primary care services delivered by nurses are of equal or even better quality compared to those provided by physicians, increasing the proportion of nurses in the practice workforce may also lead to better health outcomes and higher levels of patient satisfaction (Laurant et al. 2005, 2018; Martínez‐González et al. 2014). However, the productivity of practices may be limited by the availability of staff as practices in many areas face shortages in the supply of nurses.

The high optimal ratio of nurses to other clinical staff suggested by our production function estimates also highlights that, given the productivity of each type of staff, nurses are underpaid compared to other staff types in primary care. While the pre‐tax income of GPMS contractor GPs, the largest group of all GPs, increased from £117,300 in 2020 to £164,184 in 2023, the average salary of band 6 nurses, the predominant employment band for practice nurses, only increased from £34,250 to £37,577, significantly lower than inflation or increases in UK average earnings over the same period (K. C. Jones et al. 2024; Curtis and Burns 2020; ONS 2023, 2024). This is unlikely to be sustainable and is detrimental to nurse recruitment, retention, and morale (Anderson et al. 2021). The MRTS between DPC staff and nurses also suggests that for similar levels of cost, nurses have much higher productivity than other DPC staff. The production of care by small to medium‐sized practices does not increase as much as in larger practices from an additional DPC staff member. This finding confirms the concerns that the Additional Role Reimbursement Scheme (ARRS) may only benefit very large practices, hence may increase inequality in primary care due to correlation between practice size and other practice and population characteristics including rurality and deprivation (Hutchinson et al. 2023; B. Jones et al. 2024; Loke and Lee 2024). It is also important to note that the estimated optimal ratio of GPs to other practice staff for appointments provision is different from the optimal ratio for patient coverage, which requires a more balanced composition of clinical staff. Practices should consider this trade‐off when structuring their workforce, taking into account characteristics of the population. For example, a higher non‐GP to GP ratio may be more suitable for practices serving older populations who on average require more appointments.

While production functions have long been used to assess efficiency and productivity in primary care, most previous research suffers from inconsistent definitions of output, partly due to the lack of appointment data (Neri et al. 2022). Previously examined utilization data only cover a small proportion of practices in England and may not be representative of the overall dispersion in practice size. In this paper, we employed a flexible model particularly suited for analysing the scale and skill‐mix within primary care practices, thereby ensuring a comprehensive examination of various staffing configurations and their impacts on costs and the provision of care. Our dataset covers over 99% of primary care practices in England, fully capturing the variations in practice size in terms of staffing levels and other practice characteristics. This breadth and diversity in data enable a robust analysis of the effects of scale on primary care production, ensuring the generalizability and applicability of our findings. Another key strength that distinguishes this study from previous research on the effect of practice size on outputs is that size is measured by workforce rather than patient list size, which does not necessarily represent the productivity of the practice.

### 4.3 Limitations

This study does, however, have some limitations. First, the model assumes that the demand for appointments is equal to or greater than their supply, that is, the recorded appointment volume represents the full capacity of a practice given its workforce, skill‐mix, and characteristics. Although this assumption is likely to be true for many practices in the context of the ongoing workforce crisis in UK primary care, the productivity of the most well‐staffed practices could be underestimated if demand is lower than maximum possible productivity (Economist Impact 2023). Therefore, the true extent to which practices benefit from larger scale, that is, the slope of marginal effects, may be higher than shown in this paper.

Second, all individual appointments are assumed to be of equal value, without being adjusted for duration, purpose or quality due to the lack of practice level national data for these measures. The large literature examining the relationship between activity volumes and quality of healthcare shows mixed results, although a modest positive correlation between the quality of care and the duration of consultation was noted (Chen et al. 2009; Smith et al. 2004). Aggregated data show no evidence of trade‐off between appointment duration and volume, as the proportions of appointments taking no more than 5 min and 6–10 min have been decreasing (from 19.04% in 2022 to 15.60% in 2024 and from 18.85% to 17.67%, respectively) while the appointment volume has been increasing (NHS Digital 2024c). There may be a trade‐off between face‐to‐face appointments and other appointment modes, however. We are also unable to observe continuity of care, as the appointment data does not identify individual patients or professionals. We do observe that the ratio of marginal effect on timely appointments to the marginal effect on total appointments decreases slightly with practice size, meaning the increase in marginal production does not automatically lead to quicker access.

Third, we did not distinguish the specific role or the level of experience of the staff member within each category of staff. Using more granular workforce data with our flexible specifications would risk over‐complicating the model and is unlikely to produce meaningful and interpretable results. However, as noted in the previous section, the distribution of GP trainees is not uniform across practices, and the roles of DPC staff, the level of experience, and the contract type of GPs all vary with practice characteristics and practice size. Although the overall effect of these workforce characteristics on productivity is not clear, their omission may bias the results.

Our findings indicate that national policy encouraging primary care practices to operate at scale offers a promising opportunity to increase the availability of appointments, as additional clinical staff at larger primary care organizations provide more additional appointments than those at smaller ones. Almost all primary care practices observed in our data are smaller than the proposed primary care network size of 35,000 and we do not observe any decreasing marginal products over the current range of practice size, suggesting significant potential for improving patient access through further collaboration and expansion of practices (NHS and England 2016). However, determining the optimal practice size requires a balanced consideration of appointment availability, patient satisfaction with various aspects of primary care services, and the trade‐offs between different clinical outcomes. Working at scale may also have implications for performance of primary care practices via changes in the interactions between and the incentives of staff (Encinosa et al. 2007; Gravelle et al. 2022), which need to be traded off against the potential to increase appointment capacity.

## Funding

T.Z. is funded by a NIHR School for Primary Care Research PhD Studentship. RM and MS receive funding from the NIHR Applied Research Collaboration Greater Manchester, and MS is an NIHR Senior Investigator. The views expressed are those of the authors and not necessarily those of the NIHR or the Department of Health and Social Care.

## Conflicts of Interest

The authors declare no conflicts of interest.

## Supporting information


Supporting Information S1

## Data Availability

The data that support the findings of this study are available in NHS Digital at https://digital.nhs.uk/. These data were derived from the following resources available in the public domain: ‐ Appointments in General Practice, https://digital.nhs.uk/data‐and‐information/publications/statistical/appointments‐in‐general‐practice ‐ General Practice Workforce, https://digital.nhs.uk/data‐and‐information/publications/statistical/general‐and‐personal‐medical‐services.
