# Supplementary material for: Scale, Skill‐Mix, and Access Implications of the Production of Appointments by Primary Care Practices in England
Source: Health Econ. 2025 Nov 20;35(3):423–38. doi: 10.1002/hec.70064 (PMC12862127; doi:10.1002/hec.70064)
Supplement: Supplementary file 1 — Supporting Information S1 [file HEC-35-423-s001.docx]

## Appendix 1

Table A 1 Estimated production functions

|  | Total appointments | Two-day appointments | Weighted patients |  | GP appointments | GP two-day appointments |  | Other appointments | Other two-day appointments |
| --- | --- | --- | --- | --- | --- | --- | --- | --- | --- |
| main |  |  |  |  |  |  |  |  |  |
| Admin | 0.047578^***^ | 0.044858^***^ | 0.054371^***^ | Admin | 0.033823^***^ | 0.036189^***^ | Admin | 0.053168^***^ | 0.046517^***^ |
|  | (0.002979) | (0.003432) | (0.003204) |  | (0.002777) | (0.003402) |  | (0.003236) | (0.004451) |
| Admin^2^ | -0.000459^***^ | -0.000322^**^ | -0.000452^***^ | Admin^2^ | -0.000395^***^ | -0.000416^***^ | Admin^2^ | -0.000724^***^ | -0.000522^***^ |
|  | (0.000090) | (0.000101) | (0.000102) |  | (0.000077) | (0.000094) |  | (0.000092) | (0.000114) |
| Admin^3^ | 0.000003^***^ | 0.000003^***^ | 0.000003^***^ | Admin^3^ | 0.000003^***^ | 0.000003^***^ | Admin^3^ | 0.000003^***^ | 0.000003^***^ |
|  | (0.000000) | (0.000001) | (0.000001) |  | (0.000000) | (0.000001) |  | (0.000001) | (0.000001) |
| Non-GP | 0.045735^***^ | 0.038436^***^ | 0.014023^***^ | Qualified GP | 0.163965^***^ | 0.151295^***^ | Nurse | 0.153159^***^ | 0.158911^***^ |
|  | (0.004324) | (0.004894) | (0.003636) |  | (0.007348) | (0.009122) |  | (0.006889) | (0.011468) |
| Non-GP^2^ | -0.000927^***^ | -0.000608^*^ | -0.000273 | Qualified GP^2^ | -0.004601^***^ | -0.004562^***^ | Nurse^2^ | -0.007258^***^ | -0.006249^***^ |
|  | (0.000269) | (0.000275) | (0.000186) |  | (0.000766) | (0.000847) |  | (0.000738) | (0.001221) |
| Non-GP^3^ | 0.000008^***^ | 0.000006^***^ | 0.000003^*^ | Qualified GP^3^ | 0.000071^***^ | 0.000076^***^ | Nurse^3^ | 0.000107^***^ | 0.000085^***^ |
|  | (0.000002) | (0.000002) | (0.000001) |  | (0.000012) | (0.000013) |  | (0.000012) | (0.000020) |
| GP | 0.074070^***^ | 0.080483^***^ | 0.074798^***^ | GP Trainee | 0.076250^***^ | 0.085587^***^ | DPC | 0.063339^***^ | 0.070706^***^ |
|  | (0.004146) | (0.005229) | (0.004381) |  | (0.006842) | (0.008635) |  | (0.005936) | (0.007961) |
| GP^2^ | -0.001596^***^ | -0.001862^***^ | -0.001716^***^ | GP Trainee^2^ | -0.004018^**^ | -0.004787^**^ | DPC^2^ | -0.002702^***^ | -0.002412^**^ |
|  | (0.000304) | (0.000375) | (0.000321) |  | (0.001259) | (0.001481) |  | (0.000635) | (0.000735) |
| GP^3^ | 0.000021^***^ | 0.000026^***^ | 0.000023^***^ | GP Trainee^3^ | 0.000109^*^ | 0.000133^*^ | DPC^3^ | 0.000038^***^ | 0.000041^***^ |
|  | (0.000004) | (0.000005) | (0.000005) |  | (0.000046) | (0.000060) |  | (0.000006) | (0.000008) |
| Admin$\times$NonGP | -0.000243 | -0.000417^*^ | -0.000289^*^ | Admin$\times$Trainee | 0.000122 | -0.000158 | Admin$\times$Nurse | -0.000028 | -0.000207 |
|  | (0.000159) | (0.000162) | (0.000140) |  | (0.000328) | (0.000382) |  | (0.000259) | (0.000288) |
| Admin$\times$GP | -0.000695^***^ | -0.000802^***^ | -0.000819^***^ | Admin$\times$Qualified | -0.000687^**^ | -0.000718^**^ | Nurse$\times$DPC | -0.000238 | -0.000434 |
|  | (0.000185) | (0.000198) | (0.000227) |  | (0.000225) | (0.000247) |  | (0.000549) | (0.000796) |
| GP$\times$NonGP | 0.000225 | 0.000273 | 0.000565^*^ | Qualified$\times$Trainee | -0.002894^**^ | -0.002183 | Admin$\times$DPC | -0.000092 | -0.000432^*^ |
|  | (0.000180) | (0.000217) | (0.000263) |  | (0.001123) | (0.001277) |  | (0.000195) | (0.000220) |
| Need index | -0.183718^***^ | -0.199066^***^ |  | Need index | -0.256239^***^ | -0.177540^**^ | Need index | -0.124089^*^ | -0.221199^*^ |
|  | (0.040777) | (0.053473) |  |  | (0.045525) | (0.060511) |  | (0.053603) | (0.088944) |
| Urban | -0.012557 | -0.005345 | -0.021245 | Urban | -0.001265 | 0.001015 | Urban | -0.019353 | -0.010392 |
|  | (0.010500) | (0.014736) | (0.010931) |  | (0.012484) | (0.016704) |  | (0.013914) | (0.023515) |
| HHI |  |  | -0.000861^**^ |  |  |  |  |  |  |
|  |  |  | (0.000265) |  |  |  |  |  |  |
| Constant | 7.456845^***^ | 6.858818^***^ | 8.174905^***^ | Constant | 6.799881^***^ | 6.379465^***^ | Constant | 6.692427^***^ | 5.867511^***^ |
|  | (0.044215) | (0.057044) | (0.020572) |  | (0.048366) | (0.063927) |  | (0.060999) | (0.096166) |
|  |  |  |  |  |  |  |  |  |  |

$N=147576$; Standard errors in parentheses; ^*^ *p* < 0.05, ^**^ *p* < 0.01, ^***^ *p* < 0.001

Table A 2 Marginal effects of input variables on monthly appointment volume, evaluated for practices of different sizes measured by staffing levels of either administrative or the main type of staff input

| Practice size as measured by: | Administrative staff | | | GPs | | |
| --- | --- | --- | --- | --- | --- | --- |
|  | 25th | 50th | 75th | 25th | 50th | 75th |
| Total appointments |  |  |  |  |  |  |
| Admin | 132.62 | 138.22 | 144.73 | 134.02 | 144.13 | 155.17 |
|  | (9.26) | (9.31) | (8.84) | (9.35) | (8.12) | (7.50) |
| Non-GP | 138.76 | 152.10 | 170.81 | 118.60 | 137.76 | 164.54 |
|  | (14.78) | (12.57) | (10.31) | (10.83) | (10.84) | (11.25) |
| GP | 208.35 | 223.63 | 242.99 | 218.49 | 220.07 | 217.82 |
|  | (20.19) | (17.66) | (14.36) | (12.96) | (11.33) | (10.54) |
| Two-day appointments |  |  |  |  |  |  |
| Admin | 58.89 | 62.90 | 68.84 | 70.06 | 74.86 | 79.63 |
|  | (10.36) | (9.18) | (7.36) | (7.49) | (6.39) | (5.33) |
| Non-GP | 75.96 | 78.29 | 81.40 | 48.39 | 57.55 | 70.49 |
|  | (23.03) | (18.72) | (13.32) | (6.69) | (6.74) | (7.00) |
| GP | 125.47 | 130.82 | 137.79 | 118.18 | 118.62 | 116.48 |
|  | (21.73) | (18.03) | (13.79) | (9.09) | (8.10) | (6.58) |
| Weighted patient |  |  |  |  |  |  |
| Admin | 298.95 | 322.76 | 355.90 | 319.99 | 344.57 | 371.09 |
|  | (18.50) | (18.92) | (19.66) | (27.02) | (23.08) | (19.02) |
| Non-GP | 106.89 | 112.35 | 117.71 | 53.65 | 74.54 | 107.00 |
|  | (25.09) | (23.18) | (22.63) | (22.95) | (22.09) | (22.14) |
| GP | 419.86 | 455.70 | 503.05 | 441.78 | 442.41 | 433.62 |
|  | (47.57) | (40.49) | (30.77) | (27.38) | (24.69) | (24.17) |
|  |  |  |  |  |  |  |
| Practice size as measured by: | Administrative staff | | | Qualified GP | | |
| Total appointments with GP |  |  |  |  |  |  |
| Admin | 47.50 | 47.07 | 45.81 | 40.56 | 46.74 | 54.47 |
|  | (3.39) | (3.24) | (3.01) | (4.33) | (4.03) | (3.75) |
| GP trainee | 88.53 | 97.75 | 111.01 | 98.20 | 110.07 | 122.99 |
|  | (8.86) | (8.22) | (8.13) | (9.81) | (8.79) | (8.88) |
| Qualified GP | 210.10 | 224.40 | 243.04 | 207.63 | 228.01 | 250.01 |
|  | (8.91) | (7.79) | (6.64) | (8.23) | (8.42) | (8.82) |
| Two-day appointments with GP |  |  |  |  |  |  |
| Admin | 33.24 | 33.16 | 32.61 | 29.54 | 33.48 | 38.23 |
|  | (2.77) | (2.68) | (2.55) | (3.55) | (3.32) | (3.07) |
| GP trainee | 70.45 | 76.71 | 85.38 | 71.76 | 81.08 | 92.00 |
|  | (7.29) | (6.86) | (6.89) | (7.98) | (7.36) | (7.53) |
| Qualified GP | 126.98 | 135.90 | 147.53 | 130.00 | 139.01 | 147.44 |
|  | (6.69) | (6.04) | (5.51) | (6.89) | (7.07) | (7.09) |
|  |  |  |  |  |  |  |
| Practice size as measured by: | Administrative staff | | | Nurse | | |
| Total appointments with other practice staff |  | | |  | | |
| Admin | 79.11 | 82.31 | 83.89 | 63.42 | 71.93 | 85.74 |
|  | (3.42) | (3.76) | (4.08) | (3.44) | (3.43) | (3.54) |
| DPC | 82.97 | 95.56 | 113.78 | 83.92 | 94.78 | 112.21 |
|  | (6.96) | (7.59) | (9.03) | (7.21) | (7.96) | (9.43) |
| Nurse | 194.91 | 225.86 | 271.24 | 253.87 | 260.88 | 262.55 |
|  | (9.61) | (9.25) | (9.21) | (9.27) | (10.01) | (10.79) |
| Two-day appointments with other practice staff |  |  |  |  |  |  |
| Admin | 28.14 | 29.25 | 30.18 | 22.82 | 25.88 | 30.96 |
|  | (2.10) | (2.25) | (2.39) | (1.93) | (1.97) | (2.11) |
| DPC | 39.83 | 43.95 | 49.57 | 36.01 | 40.75 | 48.57 |
|  | (4.52) | (4.62) | (4.99) | (4.33) | (4.62) | (5.25) |
| Nurse | 86.05 | 97.38 | 113.96 | 102.64 | 107.73 | 113.03 |
|  | (6.07) | (5.92) | (5.99) | (6.07) | (6.57) | (7.10) |
|  |  |  |  |  |  |  |

Note: Standard errors in parentheses.

We test the following null hypotheses (definition see main text):

$$H_{0}:\frac{\partial E(Y_{i}|\boldsymbol{X}_{\boldsymbol{i}})}{\partial x_{m}}│_{V_{Q_{i}}^{{75}^{th}}}-\frac{\partial E\left( Y_{i} | \boldsymbol{X}_{\boldsymbol{i}} \right)}{\partial x_{m}}│_{V_{Q_{i}}^{{25}^{th}}}=0,$$

The numbers shown in the table below are the differences between the marginal effects evaluated at 25^th^ and 75^th^ percentile of administrative staff and the main type of input. The number in parenthesis is the Wald test $\chi^{2}$ statistics with significance level indicated with *.

Table A 3 Difference in marginal effects evaluated at 25th and 75th percentiles of practice size

|  | Admin 25^th^ vs 75^th^ | GP 25^th^ vs 75^th^ |
| --- | --- | --- |
| Total appointments |  |  |
| Admin | 12.104*** (3.2375) | 21.146*** (3.3924) |
| Non-GP | 32.049*** (3.6914) | 45.940*** (9.8718) |
| GP | 34.644*** (4.0700) | -0.667 (-0.0569) |
| Two-day appointments |  |  |
| Admin | 12.104*** (3.2375) | 21.146*** (3.3924) |
| Non-GP | 32.049*** (3.6914) | 45.940*** (9.8718) |
| GP | 34.644*** (4.0700) | -0.667 (-0.0569) |
| Weighted patients |  |  |
| Admin | 57.350*** (4.9545) | 51.904*** (3.4641) |
| Non-GP | 10.064 (0.7031) | 50.756*** (3.9774) |
| GP | 84.736*** (3.9514) | -9.207 (-0.3695) |
|  |  |  |
|  | Admin 25^th^ vs 75^th^ | Qualified GP 25^th^ vs 75^th^ |
| Total appointments with GP |  |  |
| Admin | -1.688 (-1.0453) | 13.907*** (7.3162) |
| GP trainee | 22.488*** (4.0201) | 24.790*** (3.0165) |
| Qualified GP | 32.938*** (7.4922) | 42.379*** (6.0387) |
| Two-day appointments with GP |  |  |
| Admin | -0.625 (-0.4662) | 8.687*** (6.1617) |
| GP trainee | 14.928*** (3.3645) | 20.248*** (3.2616) |
| Qualified GP | 20.551*** (6.2981) | 17.430*** (3.6619) |
|  |  |  |
|  | Admin 25^th^ vs 75^th^ | Nurse 25^th^ vs 75^th^ |
| Total appointments with other staff |  |  |
| Admin | 4.778** (2.5529) | 22.316*** (15.0295) |
| DPC | 30.809*** (7.1361) | 28.297*** (7.5009) |
| Nurse | 76.327*** (15.2726) | 8.675* (1.7192) |
| Two-day appointments with other staff |  |  |
| Admin | 2.039** (2.1616) | 8.133*** (10.0337) |
| DPC | 9.731*** (4.5869) | 12.558*** (6.0243) |
| Nurse | 27.917*** (9.8670) | 10.390*** (3.2300) |
|  |  |  |

$\chi^{2}$ statistics in parentheses; ^*^ *p* < 0.05, ^**^ *p* < 0.01, ^***^ *p* < 0.001

Figure A 1 Marginal effects on two-day appointments evaluated at the 10th to 90th percentile of admin staffing level

Figure A 2 Marginal effects evaluated at the 10th to 90th percentile of GP staffing level


Figure A 3 Marginal effects on GP appointments evaluated at the 10th to 90th percentile of qualified GP

## Appendix 2

Table A 4 Models controlling for additional fixed variables, region, and sub-ICB locations

|  | (1) | (2) | (3) | (4) |
| --- | --- | --- | --- | --- |
| Total appointments | Main analysis | With more fixed var | Including region | Including sub-ICB |
|  |  |  |  |  |
| Admin | 0.047578^***^ | 0.046840^***^ | 0.046884^***^ | 0.048115^***^ |
|  | (0.002979) | (0.002953) | (0.002957) | (0.002988) |
| Admin^2^ | -0.000459^***^ | -0.000450^***^ | -0.000455^***^ | -0.000496^***^ |
|  | (0.000090) | (0.000091) | (0.000091) | (0.000093) |
| Admin^3^ | 0.000003^***^ | 0.000003^***^ | 0.000003^***^ | 0.000003^***^ |
|  | (0.000000) | (0.000000) | (0.000000) | (0.000000) |
|  |  |  |  |  |
| Non-GP | 0.045735^***^ | 0.045997^***^ | 0.045321^***^ | 0.045483^***^ |
|  | (0.004324) | (0.004120) | (0.004345) | (0.004378) |
| Non-GP^2^ | -0.000927^***^ | -0.000919^***^ | -0.000950^***^ | -0.000952^***^ |
|  | (0.000269) | (0.000253) | (0.000263) | (0.000260) |
| Non-GP^3^ | 0.000008^***^ | 0.000008^***^ | 0.000008^***^ | 0.000008^***^ |
|  | (0.000002) | (0.000002) | (0.000002) | (0.000002) |
|  |  |  |  |  |
| GP | 0.074070^***^ | 0.074731^***^ | 0.075031^***^ | 0.073102^***^ |
|  | (0.004146) | (0.004150) | (0.004152) | (0.004149) |
| GP^2^ | -0.001596^***^ | -0.001598^***^ | -0.001622^***^ | -0.001514^***^ |
|  | (0.000304) | (0.000304) | (0.000310) | (0.000294) |
| GP^3^ | 0.000021^***^ | 0.000021^***^ | 0.000022^***^ | 0.000020^***^ |
|  | (0.000004) | (0.000004) | (0.000004) | (0.000004) |
|  |  |  |  |  |
| Admin$\times$NonGP | -0.000243 | -0.000245 | -0.000214 | -0.000175 |
|  | (0.000159) | (0.000152) | (0.000154) | (0.000157) |
| Admin$\times$GP | -0.000695^***^ | -0.000683^***^ | -0.000695^***^ | -0.000671^***^ |
|  | (0.000185) | (0.000186) | (0.000183) | (0.000180) |
| GP$\times$NonGP | 0.000225 | 0.000177 | 0.000199 | 0.000110 |
|  | (0.000180) | (0.000173) | (0.000174) | (0.000178) |
|  |  |  |  |  |
| Need index | -0.183718^***^ | -0.177494^***^ | -0.090827^*^ | -0.030881 |
|  | (0.040777) | (0.041666) | (0.041491) | (0.041043) |
| Urban | -0.012557 | -0.020035 | -0.017526 | -0.014601 |
|  | (0.010500) | (0.013678) | (0.013641) | (0.013355) |
| IMD |  | -0.000661 | 0.000369 | 0.000375 |
|  |  | (0.000359) | (0.000396) | (0.000425) |
| Dispensing |  | -0.004982 | -0.011769 | -0.016811 |
|  |  | (0.014351) | (0.014509) | (0.014122) |
| PMS contract |  | 0.032923^**^ | 0.023848^*^ | 0.022461 |
|  |  | (0.010301) | (0.010710) | (0.011781) |
|  |  |  |  |  |
| Safe-outstanding |  | -0.036104 | -0.036113 | -0.037997 |
|  |  | (0.064066) | (0.064267) | (0.068531) |
| Safe-inadequate |  | 0.016758 | 0.011129 | -0.007868 |
|  |  | (0.028516) | (0.027575) | (0.026815) |
| Effective-outstanding |  | -0.026557 | -0.022647 | -0.013192 |
|  |  | (0.033629) | (0.033655) | (0.033994) |
| Effective-inadequate |  | 0.031727 | 0.022616 | 0.023160 |
|  |  | (0.057856) | (0.057290) | (0.057036) |
| Caring-outstanding |  | -0.069217 | -0.066963 | -0.058729 |
|  |  | (0.035999) | (0.035487) | (0.033820) |
| Caring-inadequate |  | -0.009832 | -0.013582 | -0.015960 |
|  |  | (0.045662) | (0.046798) | (0.046506) |
| Responsive-outstanding |  | 0.004477 | 0.003122 | 0.000164 |
|  |  | (0.020050) | (0.020250) | (0.019213) |
| Responsive-inadequate |  | 0.058268^**^ | 0.060823^**^ | 0.069951^***^ |
|  |  | (0.020531) | (0.020715) | (0.020785) |
| Well-led-outstanding |  | 0.002635 | -0.002345 | -0.003040 |
|  |  | (0.025428) | (0.025150) | (0.025309) |
| Well-led-inadequate |  | -0.056550 | -0.059549 | -0.041118 |
|  |  | (0.029907) | (0.030752) | (0.029400) |
| Overall-outstanding |  | 0.016109 | 0.024421 | 0.016438 |
|  |  | (0.042754) | (0.042507) | (0.041833) |
| Overall-inadequate |  | 0.016590 | 0.026304 | 0.029122 |
|  |  | (0.054269) | (0.054049) | (0.053251) |
| CQC-missing |  | 0.030923 | 0.030229 | 0.026557 |
|  |  | (0.023510) | (0.022894) | (0.021937) |
|  |  |  |  |  |
| constant | 7.456845^***^ | 7.464544^***^ | 7.403057^***^ | 7.456326^***^ |
|  | (0.044215) | (0.044552) | (0.046645) | (0.055380) |
| N | 1.48e+05 | 1.48e+05 | 1.48e+05 | 1.48e+05 |

Standard errors in parentheses. Region, sub-ICB location, and month dummy variables are omitted from the table. ^*^ *p* < 0.05, ^**^ *p* < 0.01, ^***^ *p* < 0.001

Figure A 4 Marginal effects estimated from the model including need index, rurality, IMD, dispensing status, contract type, CQC, region, and sub-ICB location

## Appendix 3

We examined the General Practice Patient Survey results from 2019, the last pre-pandemic wave, and 2023, the midpoint of our two-year study period, and compared the responses to two questions: (1) “Overall, how would you describe your experience of your GP practice?” (2) “Overall, how would you describe your experience of making an appointment?”. Descriptive statistics of responses to the two questions are shown in the table below. The average proportions of patients satisfied with their overall experience and patients satisfied with appointment booking have both decreased significantly, from 0.851 and 0.704 to 0.740 and 0.573, respectively. Out of our sample of 6,149 practices, 5,281 had lower overall satisfaction, and 5,056 practices had lower satisfaction with making an appointment. This shows that the majority of practices have been struggling to keep up with the growing demand of GP services and to provide a sufficient number of appointments to their patients.

Table A 5 Patient satisfaction rate from the General Practice Patient Survey, 2019 vs 2023

|  | Mean | SD | p10 | p25 | Median | p75 | p90 |
| --- | --- | --- | --- | --- | --- | --- | --- |
| Overall satisfaction 2019 | .836 | .097 | 0.702 | .777 | .851 | .91 | .949 |
| Overall satisfaction 2023 | .728 | .133 | 0.547 | .64 | .74 | .827 | .894 |
| Making appointment 2019 | .695 | .145 | 0.497 | .599 | .704 | .803 | .878 |
| Making appointment 2023 | .572 | .165 | 0.355 | .452 | .573 | .69 | .791 |

Figure A 5 Marginal effects excluding practices whose patient satisfaction regarding making an appointment did not drop

Table A 6 Models with lagged input variables

|  | (1) | (2) | (3) |
| --- | --- | --- | --- |
| Total appointments | Main analysis | $t-1$ | $t-3$ |
| total |  |  |  |
| Admin | 0.047578^***^ | 0.047945^***^ | 0.048440^***^ |
|  | (0.002979) | (0.002991) | (0.003009) |
| Admin^2^ | -0.000459^***^ | -0.000464^***^ | -0.000469^***^ |
|  | (0.000090) | (0.000090) | (0.000089) |
| Admin^3^ | 0.000003^***^ | 0.000003^***^ | 0.000003^***^ |
|  | (0.000000) | (0.000000) | (0.000000) |
|  |  |  |  |
| Non-GP | 0.045735^***^ | 0.045642^***^ | 0.045519^***^ |
|  | (0.004324) | (0.004320) | (0.004349) |
| Non-GP^2^ | -0.000927^***^ | -0.000915^***^ | -0.000881^**^ |
|  | (0.000269) | (0.000270) | (0.000272) |
| Non-GP^3^ | 0.000008^***^ | 0.000008^***^ | 0.000008^***^ |
|  | (0.000002) | (0.000002) | (0.000002) |
|  |  |  |  |
| GP | 0.074070^***^ | 0.073654^***^ | 0.073233^***^ |
|  | (0.004146) | (0.004189) | (0.004282) |
| GP^2^ | -0.001596^***^ | -0.001586^***^ | -0.001610^***^ |
|  | (0.000304) | (0.000306) | (0.000315) |
| GP^3^ | 0.000021^***^ | 0.000021^***^ | 0.000021^***^ |
|  | (0.000004) | (0.000004) | (0.000004) |
|  |  |  |  |
| Admin$\times$NonGP | -0.000243 | -0.000260 | -0.000295^*^ |
|  | (0.000159) | (0.000155) | (0.000150) |
| Admin$\times$GP | -0.000695^***^ | -0.000699^***^ | -0.000692^***^ |
|  | (0.000185) | (0.000186) | (0.000185) |
| GP$\times$NonGP | 0.000225 | 0.000251 | 0.000298 |
|  | (0.000180) | (0.000176) | (0.000174) |
|  |  |  |  |
| Need index | -0.183718^***^ | -0.185880^***^ | -0.191270^***^ |
|  | (0.040777) | (0.040777) | (0.040992) |
| Urban | -0.012557 | -0.012789 | -0.012897 |
|  | (0.010500) | (0.010549) | (0.010708) |
|  |  |  |  |
| _cons | 7.456845^***^ | 7.523931^***^ | 7.628799^***^ |
|  | (0.044215) | (0.044165) | (0.044301) |
| N | 147576 | 141427 | 129129 |

Standard errors in parentheses; ^*^ *p* < 0.05, ^**^ *p* < 0.01, ^***^ *p* < 0.001

Figure A 6 Marginal effects estimated using lagged (t-1 and t-3) workforce variables

## Appendix 4

The marginal effects of the three GP inputs (GP trainee, salaried GP, and GP partner) are shown in the top row of the figure below. The marginal effects of GP trainee and qualified GP (including both salaried and partners) estimated in the main analyses are shown in the second row for comparison.

Figure A 7 Marginal effects of three GP inputs (trainee, salaried, and partner) on total GP appointments

Note: The marginal effects of GP trainees and qualified GPs (including both salaried and partners) estimated from our main analyses are shown in the second row for comparison.

Figure A 8 Marginal effects of three GP inputs (trainee, salaried, and partner) on GP two-day appointments

Note: The marginal effects of GP trainees and qualified GPs (including both salaried and partners) estimated from our main analyses are shown in the second row for comparison.

Figure A 9 Marginal effects of DPC, band 6 nurse, and band 7 nurse on total appointments with other staff

Note: The marginal effects of DPC and Nurse estimated from the main analyses are shown in the second row for comparison.

Figure A 10 Marginal effects of DPC, band 6 nurse, and band 7 nurse on two-day appointments with other staff

Note: The marginal effects of DPC and Nurse estimated from the main analyses are shown in the second row for comparison.

Figure A 11 Marginal effects using salary weighted workforce variables, (a)

Weighting (a): GP partners: £109,000 ; salaried GP: £86,530; GP trainees: £49,589 (weighted average of trainees in different grades)

Figure A 12 Marginal effects using salary weighted workforce variables, (b)

Weighting (b): Average estimated salary of GP partners: £140,200; salaried GP: £69,200^[[1]](#footnote-1)^; GP trainees excluded

Figure A 13 Marginal effects using salary weighted workforce variables, (c)

Weighting (c): GP partners: £109,000; salaried GP: £86,530 (midpoint of 22-23 BMA recommended salaried GP pay range); GP trainees excluded

1. https://digital.nhs.uk/data-and-information/publications/statistical/gp-earnings-and-expenses-estimates/2022-23 [↑](#footnote-ref-1)
